# House Governor or Medical Superintendent?—II

**Published:** 1892-08-20

**Authors:** 


### PASSING TOPICS.

### HOUSE GOVERNOR OR MEDICAL SUPERINTENDENT?—II.

It was once contended by a powerful professional journal in regard to hospital management that a medical institution ought to be medical "throughout," a form of argument which struck us at the time as too perilously akin to the Johnsonian sarcasm, "who drives fat oxen should himself be fat," to be capable of carrying conviction. Moreover, directly we enter upon a serious discussion of this proposition, we are met by the difficulty that to be medical "throughout" our hospitals must needs derive their means of support from medical sources. This surely was not intended by the journal in question, even were it possible, and we may safely dismiss the notion that the medical profession, notwithstanding its considerable contribution in unpaid services to the cause of charity, stands possessed of a right to an exclusive management of institutions maintained at the cost of non-professional supporters, who, unlike the profession, have no other object to serve than a philanthropic one. The question at the head of this article may therefore be discussed upon its merits, and constituted and equipped as our hospitals are as benevolent organisations, it resolves itself into the simple inquiry—is it to the advantage of the patients and consequentially of the institution which benefits them, that the chief executive authority should be in the hands of a layman or of a doctor? All else being equal, which form of government is the more likely to conserve the benevolent character of the work, and to represent most faithfully the intentions of its supporters?

It is a mere truism to assert that the medical profession is in its nature benevolent. More than that, we can all ungrudgingly bear witness to the fact that its members, as a body, are worthy of its best traditions. Yet it would be absurd to contend that motives of pure philanthropy induce an appreciable number of men to adopt the medical any more than any other profession or occupation, or that doctors and surgeons do not regard hospital work from a point of view altogether different from that occupied by the governors and subscribers. To the former a hospital is, above all else, a scientific workshop. It may be, and no doubt often is, something more than that, and we dismiss as rubbish the suggestion that a hospital is a place for reckless experimental treatment. Humanity is not capable of greater solicitude than is shown for the patients, and if we except from the calculation the separation from friends and, possibly, the association with other sufferers, neither of them an unmixed evil in every case, and both, of course, unavoidable, it may be asserted, without a tittle of exaggeration, that hospital patients receive advantages such as can seldom or never be commanded by the classes immediately above them in the social scale. But we hold to the opinion that the maintenance of this high standard of hospital work, in which philanthropy, religion, and science all have their share —a catholic as distinguished from a merely clinical work— depends upon the preservation of the non-professional element in the administration, and it is because the concentration of executive power in the hands of a medical officer must tend to diminish if it did not extinguish both the opportunities, and the activity of the lay authorities that we should regretfully witness a general recourse to it.

AGRICOLA.

